# The Evolutionary Analysis of Emerging Low Frequency HIV-1 CXCR4 Using Variants through Time—An Ultra-Deep Approach

**DOI:** 10.1371/journal.pcbi.1001022

**Published:** 2010-12-16

**Authors:** John Archer, Andrew Rambaut, Bruce E. Taillon, P. Richard Harrigan, Marilyn Lewis, David L. Robertson

**Affiliations:** 1 Faculty of Life Sciences, University of Manchester, Manchester, United Kingdom; 2 Institute of Evolutionary Biology, University of Edinburgh, Edinburgh, United Kingdom; 3 454 Life Sciences, Branford, Connecticut, United States of America; 4 British Columbia Centre for Excellence in HIV/AIDS, Vancouver, British Columbia, Canada; 5 Faculty of Medicine, University of British Columbia, Vancouver, British Columbia, Canada; 6 Pfizer Global Research and Development, Sandwich, United Kingdom; University of California San Diego, United States of America

## Abstract

Large-scale parallel pyrosequencing produces unprecedented quantities of sequence data. However, when generated from viral populations current mapping software is inadequate for dealing with the high levels of variation present, resulting in the potential for biased data loss. In order to apply the 454 Life Sciences' pyrosequencing system to the study of viral populations, we have developed software for the processing of highly variable sequence data. Here we demonstrate our software by analyzing two temporally sampled HIV-1 intra-patient datasets from a clinical study of maraviroc. This drug binds the CCR5 coreceptor, thus preventing HIV-1 infection of the cell. The objective is to determine viral tropism (CCR5 versus CXCR4 usage) and track the evolution of minority CXCR4-using variants that may limit the response to a maraviroc-containing treatment regimen. Five time points (two prior to treatment) were available from each patient. We first quantify the effects of divergence on initial read k-mer mapping and demonstrate the importance of utilizing population-specific template sequences in relation to the analysis of next-generation sequence data. Then, in conjunction with coreceptor prediction algorithms that infer HIV tropism, our software was used to quantify the viral population structure pre- and post-treatment. In both cases, low frequency CXCR4-using variants (2.5–15%) were detected prior to treatment. Following phylogenetic inference, these variants were observed to exist as distinct lineages that were maintained through time. Our analysis, thus confirms the role of pre-existing CXCR4-using virus in the emergence of maraviroc-insensitive HIV. The software will have utility for the study of intra-host viral diversity and evolution of other fast evolving viruses, and is available from http://www.bioinf.manchester.ac.uk/segminator/.

## Author Summary

Due to high data volumes, error rates, and short sequence lengths, new sequencing technologies present a new challenge for computational biology. In addition, high-depth (or ultra-deep) datasets, for example from pathogens, contain exceptionally large amounts of variation over short genomes or genomic regions. Here we present software for the processing and downstream analysis of such short-read viral sequence data. We apply the software to the analysis of two HIV-1 infected individuals who did not respond optimally to the drug maraviroc. For each patient, pyrosequence data was available for five time points. In both cases we detect distinct clusters of low-frequency drug-insensitive variants that were present prior to maraviroc treatment and effectively unmasked by the removal of the drug-sensitive HIV.

## Introduction

Sequencing platforms, such as the 454 Life Sciences' GS-FLX pyrosequencing system, has greatly parallelized the determination of nucleotide order within genetic material, resulting in the ability to produce extremely large datasets [1]. The vast numbers of short sequence segments produced (termed reads) in conjunction with intrinsic error rates associated with the sequencing platform [2], [3] pose challenging computational problems [4], [5]. Importantly, these data have the potential to provide previously unprecedented insight into the extent of pathogen variation (diversity) that exists within a single individual. This is particularly important in the detection of minority variants, for example, those associated with drug resistance [6]–[11].

To date, software has focused on eukaryotic and prokaryotic genome-scale sequencing with its associated megabase reference genomes and vast quantities of read data [5], [12]. For such studies traditional fast alignment algorithms [13]–[15] that employ flexible k-mer matching are not capable of mapping reads to a reference sequence within a reasonable time. Consequently new software tools have been developed that incorporate faster string matching techniques at the expense of dealing with variation [12], [16]–[18]. For highly variable genomes this limitation will result in data loss as reads with more than the specified numbers of mismatches, in relation to a template sequence, are discarded. This loss can occur non-randomly with reads representing minority subpopulations being less likely to be mapped to the template. For example, two distinct phenotypes of HIV-1 exist that are defined by the host coreceptor that is used during cell entry. The coreceptors involved are chemokine (C–C motif) receptor 5 (CCR5) and chemokine (C-X-C) receptor 4 (CXCR4). The location of the viral genome that determines the phenotype is the third variable (V3) loop, a highly variable region [19] located within HIV's envelope gene, *env* [20]–[22]. The most often used genomic reference sequence for HIV-1 is HXB2, a CXCR4-using virus. When mapping V3 data to HXB2, and limiting the number of mismatches allowed, reads representing CCR5 variants are more likely to be lost during mapping as a result of known amino acid changes associated with that phenotype [23], [24]. This may result in a misleading ratio of coreceptor use within an HIV-1 population, which can have consequences for drug treatment decisions [25]. Thus, for rapidly evolving viruses, such as HIV-1 [26]–[29], a limitation on the number of mismatches tolerated during read mapping is less than optimal. Modification of traditional k-mer matching approaches [13] is a more suitable approach and becomes scalable (due to the smaller genome sizes) as they allow for increased tolerance when dealing with higher levels of variation in viral 454 data [6], [11].

**Figure 1:**
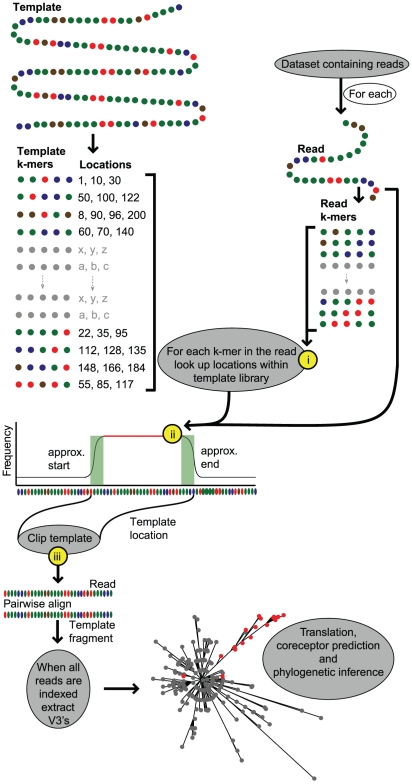
The data analysis framework. On the left hand side the preprocessing of the template sequence prior to read mapping is illustrated. The fragments titled “k-mers” are all the unique words (length = 5) within the template sequence. These are stored along with their corresponding locations. On the opposite side all k-mers of equal length, extracted from the read, are shown. The plot indicates the frequency of k-mer matches across the template sequence for a single read. Grey boxes indicate processing events that take place within the framework. The yellow circles indicate optimization steps: (i) only exact k-mer matches used (ii) a heuristic alignment not constructed from the k-mer matching (just the k-mer match frequencies are plotted) and (iii) only the appropriate region of the template is pairwise aligned to the read.

Prior to any evolutionary study reads must be accurately mapped and aligned. Our software performs these tasks as well as subsequent tropism testing, phylogenetic tree inference and visualization (Fig. 1). We demonstrate the software's underlying framework in order to quantify the effects of divergence on the mapping of reads to a template sequence. In addition to unbiased mapping of data, a reduction of divergence between reads and template is favorable for the removal of platform dependent insertions. Characteristically with 454 data there is a high rate of insertion error associated with the chemistry involved during the pyrosequencing process [2], [3]. Failure to remove such insertions can result in a further loss of usable data when translations are required during downstream analysis.

We apply our software to temporally sampled 454 datasets from two HIV-1 infected individuals in order to characterize the emergence of low frequency CXCR4-using variants following treatment with an HIV entry-inhibitor drug, the CCR5 antagonist maraviroc. As the drug will not directly impact on viruses using the CXCR4 coreceptor [30], patients are screened for their presence prior to treatment [25], [31]. The aim is to distinguish a viral population that is exclusively CCR5 tropic (R5) from a viral population including either dual-mixed, DM, (R5 and exclusively CXCR4-using [X4]), or R5 and dual-tropic viruses (those that can use both CCR5 and CXCR4 [R5X4]). Note, we refer to both X4 and R5X4 tropic viruses as CXCR4-using. This application of our software demonstrates that sequence data generated from the 454 platform – in conjunction with coreceptor prediction tests based on HIV's V3 region [23], [24], [32] – permits the quantification and evolutionary analysis of HIV-1 tropism present at low frequencies within a sample more effectively than could be achieved using standard population sequencing technologies [6], [11]. Our software will also have utility for studying the within-host diversity of other fast evolving viruses.

## Methods

### Datasets

Samples for pyrosequencing were obtained from two HIV-1 infected males, patients D and E, both of whom were treatment naïve and enrolled in the QD arm of the A4001026 study [31], were infected with subtype B virus and received maraviroc once daily together with zidovudine and lamivudine. They discontinued the study due to insufficient clinical response: patient D continued to week 2 and patient E to week 24. Both had the M184V mutation in reverse transcriptase at failure, which confers resistance to the background therapy and, in addition, patient D had M41M/L and K70K/R. Samples were collected over five time points: screening (40 days before), day 1, week 2 (day 15), week 12 (day 80) and week 16 (day 107) for patient D and screening (41 days before), day 1, week 8 (day 57), week 24 (day 162) and week 30 (day 211) for patient E.

For each sample, RNA extraction and amplification from the gp120 region of *env* was performed. The amplicons were subjected to nebulization to generate fragments of approximately 600 nucleotides. These were amplified as described in Margulies *et al.* [1]. and sequenced on the Genome Sequencer 20 (GS20, Roche Applied Sciences). Standard protocols for the generation of a library of tagged single-stranded DNA molecules were used (for details, see Margulies *et al.* [1]). The GS20 software package was used to generate the sequence files. This resulted in files containing between 14,000 and 31,000 reads (Table 1). The data is available at the NCBI Sequence Read Archive (http://www.ncbi.nlm.nih.gov/Traces/sra) under accession number SRA023641.1.

**Table 1:** Read extraction comparison.

Time point	No. of reads across entire gp120	No. of reads mapped to a dataset-specific consensus across V3	No. of reads mapped to HXB2's V3 region	Difference between dataset-specific consensus and HXB2 mapping	% data lost using HXB2	Divergence between templates (hamming)
**Patient D**						
** Screening**	30,686	8,385	6,309	2,076	24.8	0.1369
** Day 1**	28,902	8,655	6,438	2,217	25.6	0.1369
** Week 2**	28,521	8,009	5,679	2,330	29.1	0.1320
** Week 12**	23,312	6,845	5,076	1,769	25.8	0.1393
** Week 16**	14,880	3,591	2,669	922	25.7	0.1589
**Patient E**						
** Screening**	12,646	3,257	1,813	1,444	44.3	0.1736
** Day 1**	18,381	4,386	2,891	1,495	34.1	0.1589
** Week 8**	18,551	4,085	2,611	1,474	36.1	0.1418
** Week 24**	19,268	4,723	3,228	1,495	31.7	0.1840
** Week 30**	17,993	4,764	2,890	1,874	39.3	0.1711

Comparison of the number of reads extracted at each time point for patients D and E using k-mer mapping for a dataset-specific consensus and HXB2 templates. The genome coordinates 6900 to 7305 were used so as to include the V3 region and all reads spanning V3. The numbers in the first column are the total number of reads covering gp120.

### K-mer mapping

Although RNA extraction and amplification was carried out across the entire gp120 gene, the region that is required for the coreceptor prediction of HIV-1 variants is V3 [23], [24], [32]. Reads covering this region were identified using a k-mer matching process similar to the initial phase of the BLAST algorithm [13]. For a single read the location of all k-mers of size five are identified across the template sequence. If a matching region is found for the read, the frequencies of k-mer hits will be above the random level of background noise at that location (Fig. 1). For each dataset the coordinates of the template in relation to the HXB2 reference genome are 6900 to 7305. This accommodates longer reads that may span the entire V3 region. The coordinates of the V3 loop within this region are 7110 to 7217. Dataset specificity was generated within the templates using a pre-mapping to the HXB2 reference sequence for which multiple alignments are then generated within windows of size 70 (using a 20 nucleotide overlap). Within each of these alignments columns containing more than 50% gaps were removed. Consensus sequences, created for each alignment, are then appended in order to form data-specific templates to which reads are then remapped.

To explore the effects of using a consensus template on k-mer mapping, for each dataset, we compared the number of reads mapped to the data-specific template for that dataset to the number of reads mapped using HXB2 – the latter being the pre-mapping stage prior to consensus template generation. Next, we took our patient D screening dataset and introduced random variation into the consensus template in sequential steps of 2, 4 up to 26% (50 repetitions for each). After each introduction of random variation, the k-mer mapping was performed and the number of reads successfully mapped recorded. It should be noted that no precise pairwise alignment to the template sequence is generated during k-mer mapping. Instead the reads within our datasets covering the portion of the HIV-1 genome between the coordinates 6900 to 7305 are identified along with their approximate start positions. The k-mer mapping approach implicitly allows for a higher degree of tolerance in identifying such reads when compared to approaches that limit the number of mismatches [12], [16]–[18] as, although the process involves matching exact k-mers to the template at any given location, the overall frequency of k-mer hits will increase at the most likely location of the read across the template (Fig. 1). This approach, thus, does not specify an exact limitation on the number of mismatches allowed between the read itself and the template.

### Pairwise alignment

Following k-mer mapping, reads are pairwise aligned to the consensus template using Smith and Waterman [33]. Indices obtained during k-mer mapping are used to optimize the process by only aligning reads to the appropriate region. Platform dependent insertion error, which makes up the majority of non-biological error [2], [3], is accounted for by maintaining reference to the dataset specific template. Specifically, insertions relative to the template, which represents an in frame consensus sequence, are removed. The frequency at which these insertions occurred across the V3 region was recorded. The usage of a data-specific consensus sequence is important to ensure that insertions naturally existing within the population are not erroneously removed based on use of a divergent template sequence.

### Tropism prediction and phylogenetic inference

Reads spanning the V3 region of *env* were extracted, truncated, identical reads removed (frequencies were stored) and multiply aligned using Muscle [34], packaged with the software. Coreceptor prediction was performed using the 11/24/25 “charge rule” [23], [32], implemented within the software and using the PSSM web tool [24]. Sequence logos were generated for inferred R5 and CXCR4-using sequence present at each time point using the Web Logo tool [35]. Nucleotide sequences, annotated with coreceptor predictions, were used to infer evolutionary relationships by maximum likelihood using PhyML [36], packaged with the software. The HKY model of sequence evolution was used. The resulting phylogenetic trees were visualized using an integrated version of CTree [37]. Because bootstrapping is unreliable when performed on very short sequence alignments, the significance of the identified clusters within datasets representing the early time points was determined by comparing the ratio between the intra-cluster pairwise distance and the inter-cluster pairwise distance (of five random clusters) to a distribution of values obtained for 500 sets randomly assigned clusters. A low intra-cluster pairwise distance relative to the inter-cluster pairwise distance implies a robust cluster [38], [39]. Additionally clustering significance was tested using the *approximate likelihood ratio test* [40] for branches as implemented within PhyML.

### Key functions of the software

The pipeline used for processing the initial read data is available within our software (Fig. 1). Implemented in Java the executable runs on Mac OS X, Linux and Windows. All required external binaries are included within the package. The input is a FASTA formatted file containing unmapped read data. Output files are in FASTA, TXT, PDF or NEWICK format as appropriate. A summary of the key functions incorporated into the software are: (i) accurate mapping of next generation sequence data containing high amounts of variation, (ii) exportation of reads spanning user defined regions of the template, (iii) translation of reads, (iv) determination of nucleotide and/or amino acid residue frequencies, (v) generation of a consensus sequence across the entire dataset taking into account data-specific indels, thus, reducing dependency on a generic template, (vi) removal of reads based on a hamming distance from their corresponding region on the template, (vii) generation of a multiple alignment of reads spanning a particular region of the template using Muscle [34], (viii) detection and annotation of low frequency variants, (ix) inference of phylogenetic trees using PhyML [36], (x) tree label searching based on the annotation produced in viii and visualized using CTree [37], and (xi) management of bar coded data. During the scaffolding process, a number of output plots are generated to summarize the data. These include read length distributions and template coverage. The latter is portrayed in a circular plot to allow for longer templates to be displayed optimally.

## Results

### K-mer mapping

**Figure 2:**
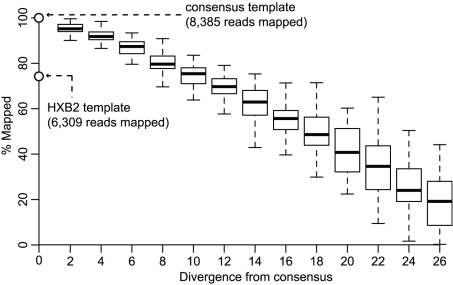
Relationship between k-mer mapping and diversity. As divergence from the consensus template increases the number of reads successfully mapped decreases. Each box and whisker (1.5 times the inter-quartile range) represents 50 repetitions of the mapping process at the level of divergence indicated on the x-axis. The bottom circle, on the y-axis, indicates the percentage of reads mapped to HXB2 in relation to the total number mapped to the consensus template (top circle). The dataset used for this comparison was patient D at screening.

For each dataset, following k-mer mapping to the consensus template, high read coverage was observed across the V3 region (Table 1). In each case when HXB2 was used as a template sequence fewer reads are mapped. For patient D the mean loss of reads is 26.4%, while for patient E it is 36.5%, the difference being due to the divergence between patients D and E's data-specific templates and HXB2 (Table 1). When random variation in sequential steps of 2 to 26% was introduced into the consensus template derived from the patient D screening dataset and k-mer mapping performed on reads from that dataset, a reduction in the number of mapped can be observed that is directly proportional to increasing divergence (Fig. 2).

### Pairwise alignment

Reads were pairwise aligned to the appropriate region (identified from the k-mer mapping step) of the data-specific consensus templates and those spanning both the start and end of the V3 region were extracted and truncated. Between 17 and 33% of reads contain at least one insertion event across the V3 region in comparison to the consensus template (Table 2). The vast majority of these insertions were observed to be singleton or dinucleotide insertions (causing a frame shift), with a mean per site frequency of 0.18%. Note, this frequency is after the sequences have been truncated. These are lower numbers than would be expected if complete reads had been included [3] as the starts and ends of the majority of the reads have been removed by the truncation step. During the alignment process such insertions were removed in order to maintain as many correctly translated V3 regions as possible.

**Table 2:** V3 region coverage.

Time point	No. of complete V3's initially extracted	No. of insertions present	No. of reads containing at least one insertion	% per site insertion frequency across the V3 region
**Patient D**				
** Screening**	2,022	489 (24.2%)	581	0.27
** Day 1**	2,081	471 (22.6%)	533	0.24
** Week 2**	2,266	655 (28.9%)	759	0.35
** Week 12**	1,609	378 (23.5%)	440	0.2
** Week 16**	908	183 (20.2%)	207	0.1
**Patient E**				
** Screening**	778	174 (22.4%)	199	0.1
** Day 1**	1,022	180 (17.6%)	216	0.1
** Week 8**	1,082	352 (32.5%)	506	0.23
** Week 24**	1,015	253 (24.9%)	387	0.17
** Week 30**	1,244	235 (18.9%)	267	0.12

The total number of reads completely spanning the V3 region (coordinates 7110 to 7217), regardless of the presence or absence of frame shift errors. The percentage containing singleton or dinucleotide insertion events across this region is displayed.

### Tropism prediction and phylogenetic inference

For both patients a high coverage of in-frame reads across the V3 region was observed at each time point (Tables 3 and 4) with many unique variants. Those reads that could not be translated correctly were discarded, resulting in the lower numbers observed in Tables 3 and 4 than those presented in table 2. When both the charge rule and PSSM tests were performed on these data, CXCR4-using variants were detected prior to treatment within both patients (Tables 3 and 4). On maraviroc treatment, for patient D, CXCR4-using virus increased to a frequency of 41% and for patient E increased to 99% at the sampling times (Tables 3 and 4). Interestingly, despite the CXCR4-using population increasing in patient D and becoming dominant in patient E on-treatment, the reduction in viral load corresponds to an order of magnitude less CXCR4-using virus than that prior to treatment.

**Table 3:** Patient D tropism predictions.

Time point	R5 (%)	CXCR4-using (%)	Viral load (copies/ml)	CXCR4-using viral load (copies/ml)	No. V3 of reads and unique reads in brackets.	CD4 (cells/mcl)
**Screening**	pre-treatment with maraviroc					
Charge Rule	93.5	6.5	668,000	43,420	1,743 (284)	31
PSSM	93.7	6.3				
**Day 1**	pre-treatment with maraviroc					
Charge Rule	86.7	13.3	673,000	89,509	1,755 (266)	29
PSSM	86.7	13.3				
**Week 2**	on-treatment with maraviroc					
Charge Rule	58.7	41.3	3,560	1,470	1,897 (196)	88
PSSM	58.8	41.2				
**Week 12**	off-treatment with maraviroc					
Charge Rule	82.1	17.9	25,300	4,528	1,344 (194)	97
PSSM	82.4	17.6				
**Week 16**	off-treatment with maraviroc					
Charge Rule	42.7	57.3	1,890	1,082	710 (106)	N/A
PSSM	42.7	57.3				

Predicted coreceptor usage, viral load, estimated proportion of population that is CXCR4-using (from % estimated by charge rule), number of V3 sequences extracted (in-frame) and CD4 cell count at different time points pre- and post-treatment for patient D.

**Table 4:** Patient E tropism predictions.

Time point	R5 (%)	CXCR4-using (%)	Viral load (copies/ml)	CXCR4-using viral load (copies/ml)	No. V3 of reads and unique reads in brackets.	CD4 (cells/mcl)
**Screening**	pre-treatment with maraviroc					
Charge Rule	97.5	2.5	476,000	11,900	651 (113)	27
PSSM	97.7	2.3				
**Day 1**	pre-treatment with maraviroc					
Charge Rule	85	15	350,000	52,500	833 (147)	12
PSSM	94.72	5.28				
**Week 8**	on-treatment with maraviroc					
Charge Rule	0.7	99.3	23,000	22,839	900 (93)	162
PSSM	60.9	39.1				
**Week 24**	on-treatment with maraviroc					
Charge Rule	0.5	99.5	5,420	5,392	1123 (153)	176
PSSM	1.4	98.6				
**Week 30**	off-treatment with maraviroc					
Charge Rule	0.4	99.6	85,700	85,357	1054 (126)	132
PSSM	3.1	96.9				

Predicted coreceptor usage, viral load, estimated proportion of population that is CXCR4-using (from % estimated by charge rule), number of V3 sequences extracted (in-frame) and CD4 cell count at different time points pre- and post-treatment for patient E.

**Figure 3:**
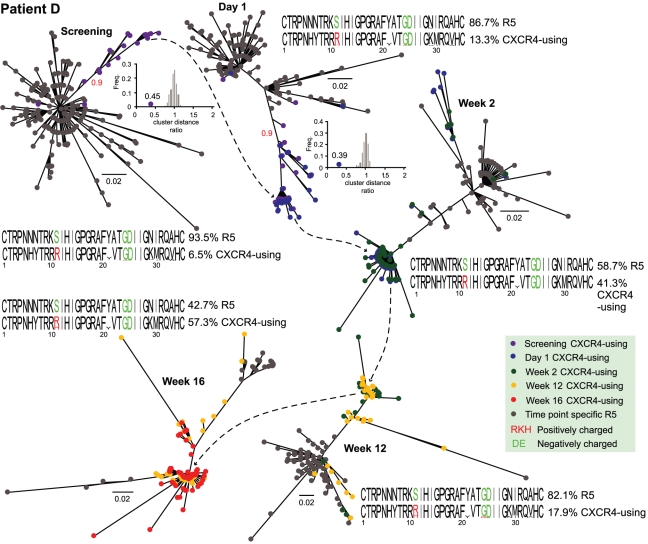
Evolutionary relationships of patient D's viral population through time. Each phylogeny shows the predicted R5 and CXCR4-using variants for the time points: screening, day 1, week 2, week 12 and week 16; only unique variants are shown. Subsequent to screening, the CXCR4-using variants from the previous time point are included for visualization purposes. Sequence logos for R5 and CXCR4-using sequences for each time point are also shown. Colors (see key) indicate sampling time in phylogenies and residue charges in sequence logos. The red numbers on the lineage separating branches at screening and day 1 indicate the branch support value from the *approximate likelihood ratio test* for the distinct CXCR4-using lineage present at these time points. The inset plots indicate the extent of the clustering present for these same lineages and time points (value next to circle on x axis) in comparison to a distribution of randomly assigned clusters; see methods for further details. The scale bar represents nucleotide substitutions per site.

**Figure 4:**
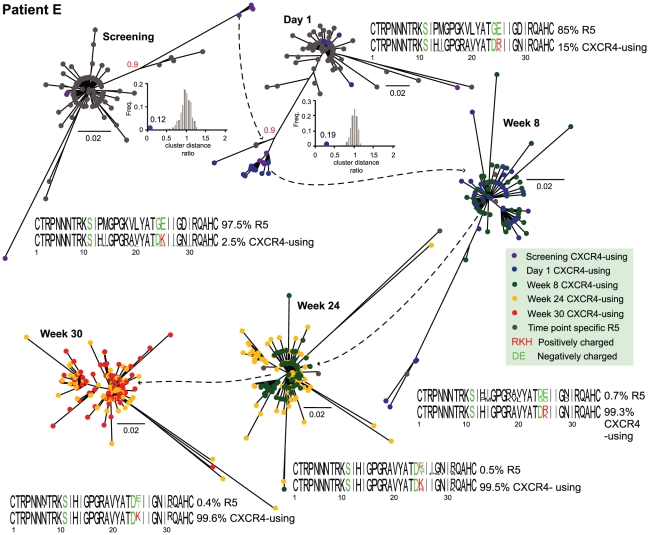
Evolutionary relationships of patient E's viral population through time. Each phylogeny shows the predicted R5 and CXCR4-using variants for the time points: screening, day 1, week 8, week 24 and week 30. See figure 3's legend for further details.

The inference of the evolutionary history for each dataset, revealed the majority of CXCR4-using variants formed a distinct cluster, present prior to maraviroc treatment and divergent to the main R5 populations present prior to treatment (Fig. 3 and. 4). The inset plots confirm the significance of each CXCR4-using cluster based on the comparison of intra- and inter-pairwise distances and confirmed using the *approximate likelihood ratio test* (Fig. 3 and 4). The sequence logos beside each phylogeny represents a comparison between the sequence characteristics of the R5 tropic and CXCR4-using variants. At each time point, key differences in charge [23], [24], [32] can be observed at sites 11 for patient D and at site 25 for patient E.

For each patient for the two time points prior to therapy when the CXCR4-using variants located within the R5 tropic clusters were investigated, they were observed to be more similar to their closely related R5 tropic counterparts than to the distinct clusters of CXCR4-using variants (Fig. 3 and 4, screening and Day 1). For patient D there are four such variants (1.1% of the CXCR4-using population), while for patient E there are ten (7% of the CXCR4-using population) prior to therapy.

**Figure 5:**
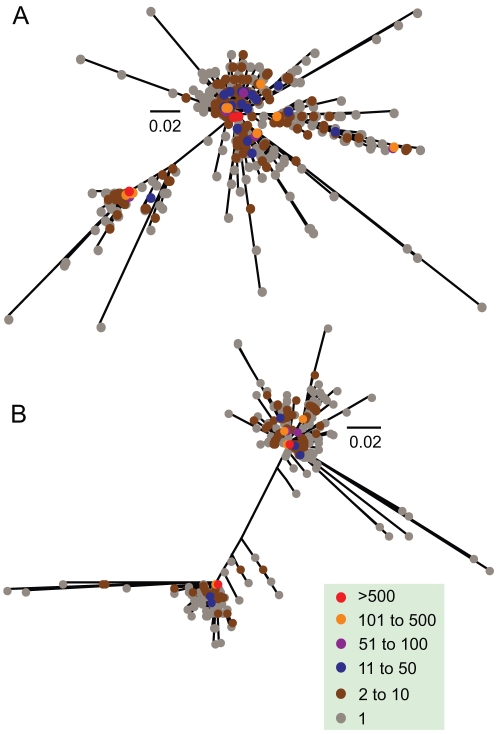
Frequency of HIV-1 variants in the phylogenetic trees. Evolutionary tree inferred from all patient D's V3 nucleotide sequences (A), and all patient E's V3 nucleotide sequences (B). Colors (see key) indicate the frequency of each sequence. The scale bar represents nucleotide substitutions per site.

It is important to note, the extent of the divergence in the phylogenetic trees is mainly due to the high number of either unique or rare variants. These variants cluster around high frequency variants within the population (Fig. 5). For example, at screening (Patient D) only ten variants make up 75% of the viral population with a single variant contributing to 23% of the population. Despite a proportion of this variation being due to sequencing error, this level of variation emerging in relatively short time periods highlights the extreme mutability of HIV.

**Figure 6:**
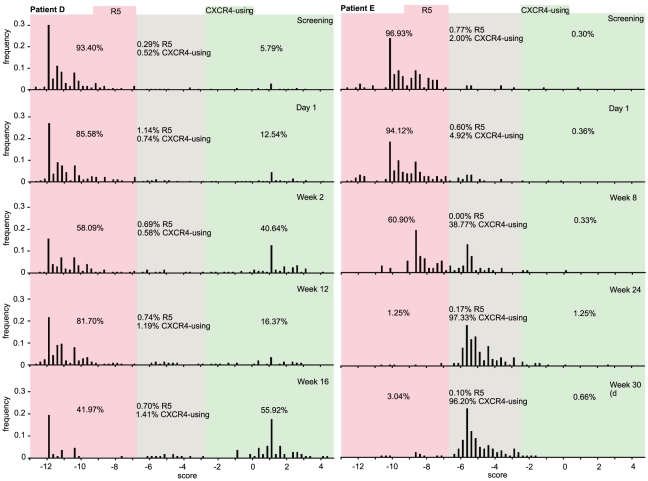
Tropism prediction. Frequency plots of PSSM scores of unique V3 sequences within each dataset. The red area indicates the region below the −6.96 threshold (R5), the green region indicates the area above the −2.88 threshold (CXCR4-using) and the grey area indicates the region between the two thresholds. The numbers within each plot area indicate the percentage of reads called as R5 or CXCR4-using for that region.

Tables 3 and 4 show the results of the PSSM test carried out on the extracted V3 sequences. In all cases, with the exception of patient E (week 8), PSSM confirms similar levels of CXCR4-using virus to those predicted by the charge rule. PSSM predicts viruses to be CXCR4-using based on scores being higher than a threshold of −2.88 (Fig. 6), and to be R5 tropic based on scores being below a threshold value of −6.96 (Fig. 6). Between these two threshold values a reliable PSSM prediction cannot be made and so composite PSSM utilizes the charge rule at sites 11 and 25 [24]. Note, we have also included site 24 in the composite prediction [23].

## Discussion

We have developed freely available software for the management and downstream analysis of pathogen sequence data. We demonstrate the utility of this software by applying it to the detection, and subsequent evolutionary analysis, of drug resistant variants within two temporally sampled patients infected with HIV-1. In our study of the V3 region we demonstrate that both the CXCR4-using viral populations, which emerge during maraviroc treatment, do not evolve *de novo*. Instead, confirming previous studies [6], [11], [41], they emerge from a pre-existing, distinct, viral subpopulation that is present prior to therapy (Fig. 3 and 4). Note, the phylogenetic analysis was repeated excluding sites 11, 24 and 25 (those used in the charge rule test), and the same divergent CXCR4-using cluster identified (and statistically supported), indicating convergent evolution is not biasing the inferences. If the R5 variants had evolved during treatment to become CXCR4-using they would be more closely related to these R5 counterparts. In addition, characteristic differences between R5 and X4 variants (specifically at sites 11, 24 or 25 in V3) are observed at screening and day 1 (Fig. 3 and 4, sequence logos).

We quantified the effects of HIV-1 mutability on the k-mer mapping process prior to downstream analysis. Using the patient D screening dataset, in conjunction with the consensus template for that data within which random mutations were introduced in sequential steps, we observed that at a divergence level of 26% just over 20% of the reads originally mapped to the unaltered consensus map successfully (Fig. 2). This demonstrates there is a direct relationship between the number of reads that are mapped successfully and the level of divergence between the data and template sequence. The usage of an inappropriate template will, thus, very probably result in the non-random loss of data, introducing an unnecessary bias. Indeed for each of our datasets, when mapped to HXB2 rather than the data-specific consensus templates, between 24 and 44% of reads covering the V3 region were not mapped as a result of divergence between the consensus templates and HXB2 which ranged form 14 to16.5% (Table 1).

When an amino acid translation step is performed, in our case for inferring reads as R5 or CXCR4-using, data loss can be further minimized by utilizing a correction procedure relative to the in-frame template sequence. Platform-dependent insertions make up the majority of sequencing error usually resulting in an over-representation of frame shifts within the reads [2], [3]. Correction based on a divergent template will result in a greater probability of complete codons being removed erroneously and therefore it is optimal to use a template that is dataset specific (Figure S1). In Tsibris *et al.*, [11] where no such correction was performed on temporally sampled data from two subtype B infected patients much of the data was removed. Within one sample a platform dependent insertion within a known homopolymeric stretch resulted in the staggering removal of 85% reads. Using a correction approach, based on an in-frame consensus template, reduces this loss greatly (Table 5).

**Table 5:** Reference template comparison.

		No reading frame correction		Data-specific consensus based frame correction	
Sample ID	No. of Reads	No. V3's	No. of V3's used after translation	No. V3's	No. of V3's used after translation
**18.00**	138,681	130,268	110,471 (80%)	138,363	132,197 (95%)
**18.02**	62,475	52,403	25,419 (41%)	61,747	54,558 (87%)
**18.16**	98,025	86,392	14,366 (15%)	97,374	73,862 (75%)
**19.00**	70,391	64,978	59,226 (84%)	69,937	66,655 (95%)
**19.02**	46,826	43,224	38,217 (82%)	46,563	43,982 (94%)
**19.17**	25,685	23,755	22,519 (88%)	25,483	24,384 (95%)

Comparison of use of a data-specific template with a published study which used HXB2 as a reference template [11]. Dataset sizes are from Tsibris *et al.*, [11]. The number of in-frame reads available for downstream analysis after correction based on a data-specific consensus is compared to the number of in-frame reads when no correction is applied.

Algorithms for the computational prediction of tropism are highly dependent on the available training datasets. In the case of PSSM, for example, the training data used in the web tool defines the threshold cutoff values (−2.88 and −6.96) used in the coreceptor prediction [24]. When data falls between the current threshold values the PSSM web tool uses the charge rule [24]. This can be misleading as seen for patient E week 8 (Fig. 6), the charge rule called 99.3% of the population as CXCR4-using based on the presence of a positive charge at site 25, while PSSM called 39.1% of the population as CXCR4-using (Table 4). For the latter only 0.3% of variants fall above the PSSM CXCR4-using threshold. The remaining 38.8% of CXCR4-using variants is based on the charge rule and not the PSSM scores. The variants that fall below the CCR5-using threshold (60.9%), despite the majority still possessing a positively charged residue at site 25, have been called based on their PSSM scores. The most likely explanation is that these variants are dual tropic and typing them as R5 is incorrect.

It is also important to consider how much CXCR4-using virus is acceptable in the context of combination therapy. At present a 2% threshold has been proposed by RH [42]. In our study both patients had greater than 2% CXCR4-using virus at screening and the CXCR4-using population was greater than 10,000 copies/ml. Interestingly, although the CXCR4-using virus is clearly present during therapy, the overall CXCR4-using plasma HIV-1 RNA was reduced during the treatment phase, presumably due to the effect of the other drugs used with maraviroc.

In conclusion, our results demonstrate that, in conjunction with appropriate software, pyrosequencing data has utility for the evolutionally analysis and detection of low frequency variants within viral populations. In our analysis we have provided a high-resolution snapshot, through temporally sampled data, of intra-patient viral diversity and evolution associated with the CCR5-antagonist maraviroc. We have also quantified the effects of viral diversity on the initial k-mer mapping of read data in relation to the correction of platform dependent insertion error. The features of the software used here can be applied to other drug susceptibility and resistance studies, within other genomic regions of HIV-1 or to other pathogen genomes.

## Supporting Information

Figure S1 Example of frame correction to a dataset specific template and HXB2. Pairwise alignment of the V3 region of the consensus template (patient D, screening) to that of HXB2 (A). Correction of the V3 sequence based on HXB2, and subsequent loss of a complete codon (B). Correction of the V3 sequence based on the data-specific template, and no codon loss (C).
(0.46 MB EPS)
